# Draft Genome Sequence Resource of *Erwinia* sp. Strain INIA01, a Phytopathogen Isolated from a Diseased Stalk of Peruvian Maize

**DOI:** 10.1128/mra.00120-23

**Published:** 2023-04-13

**Authors:** Richard Estrada, Carla L. Saldaña, Wendy Pérez-Porras, Linda Arteaga, Gabriel Martínez, Pedro Injante, Moises R. Duran, Wilian Salazar, Roberto Cosme, Carlos Poemape, Carlos I. Arbizu

**Affiliations:** a Dirección de Desarrollo Tecnológico Agrario, Instituto Nacional de Innovación Agraria (INIA), Lima, Peru; b Estación Experimental Agraria Vista Florida, Instituto Nacional de Innovación Agraria (INIA), Picsi, Ferreñafe, Peru; c Facultad de Ingeniería y Ciencias Agrarias, Universidad Nacional Toribio Rodríguez de Mendoza de Amazonas (UNTRM), Chachapoyas, Amazonas, Peru; Wellesley College

## Abstract

Here, we report the complete genome sequence of *Erwinia* sp. strain INIA01, a bacterium isolated from lesions of Zea mays from northern Peru. This genome possesses two circular replicons, a 4.2-Mb chromosome, and a 438-kb plasmid.

## ANNOUNCEMENT

The genus *Erwinia* has several plant pathogens; they distinguish themselves by their great capability to infect and spread, causing huge losses in many crops. Since these diseases are highly contagious and result in serious losses once introduced, they are regulated as a quarantine disease (1–4). An outbreak of bacterial rot in maize hectares was intercepted in Chiclayo, Peru (6.72°S, 79.77°W). Plant tissues with symptoms of soft wet stem rot and nauseous smell were collected, and the surface was cleaned with sterile distilled water. The tissues were cut under a laminar flow hood using a scalpel and serially diluted in peptone-water. The culture was done in nutrient agar plates for isolation (28°C, 48 h). The strain was characterized by biochemical tests (5, 6).

The strain isolated on nutrient agar was selected for the extraction of its genomic DNA; we used the E.Z.N.A. bacterial DNA isolation kit (Omega Bio-tek, USA) following the manufacturer’s protocol. The genomic DNA was subjected to 150-bp paired-end (PE) Illumina sequencing using the Illumina Nextera DNA Flex library preparation kit. The PE Illumina library was loaded onto the NovoSeq 6000 instrument for cluster generation and sequencing by Novogene Co. Ltd. (CA, USA). A total of 9,231,680 paired-end reads representing ~150× genome coverage was generated. Quality trimming (Phred Q of >25) was conducted with Trimmomatic v0.36 (7). *De novo* assembly was performed with Spades v 3.10.1 (8) with testing of different k-mers (from 23 to 123). SSPACE v2.0 (9) and GapCloser (10) were used for scaffolding. We used QUAST v.5.2.0 (11) for statistics of assemblies. The completeness and consistency of the assembled genome were estimated using Benchmarking Universal Single-Copy Orthologs (BUSCO) (12) and CheckM (13), showing 100% completeness. Extrachromosomal content was identified by rerunning SPAdes on the raw reads with the “plasmid” flag. Plasmids were further visually confirmed for circularity using assembly graphs constructed in Bandage v.0.8.1. (14). The genome sequence consists of 5,702,202 bp (41 scaffolds; *N*_50_, 313,858 bp); furthermore, a scaffold was identified as a plasmid with a size of 438,452 bp. The scaffolds obtained were further annotated with NCBI Prokaryotic Genome Annotation Pipeline (PGAP) and as performed using RASTtk v2.0 using default parameters (15–17). A total of 5,062 protein-coding genes were estimated from assembly. Also, we detected 73 RNAs (2 rRNAs, 64 tRNAs, and 7 noncoding RNAs [ncRNAs]) and 58 pseudogenes (Table 1).

**TABLE 1:** Quality statistics of *Erwinia* sp. INIA01

Feature	Result
Consistency (%)	
Coarse	99.4
Fine	98.2
CheckM (%)	
Completeness	100
Contamination	0.51
No. of BUSCOs	
Complete	124
Duplicated	0
Fragmented	0
Missing	0
No. of scaffolds	
≥1,000 bp	41
≥50,000 bp	21
Largest scaffold, bp	830,855
Total length, bp	5,702,202
GC (%)	55.33
*N*_50_	313,858
No. of N’s per 100 kbp	28.06
No. of CDSs^*a*^ (with protein)	5,062
No. of RNAs	76
No. of rRNAs	2
No. of tRNAs	64
No. of ncRNAs	7
No. of pseudogenes (total)	58

a CDSs, coding sequences.

An EzBioCloud BLAST analysis of the 16S rRNA gene sequence of strain INIA01 yielded the highest identity (99.1%) with the *Erwinia* sp. strain ELC0701. Based on average nucleotide identity (ANI) with GTDB-Tk v0.3.2 analysis (18, 19), our genome is closely related to *Erwinia* sp. strain Leaf5 (20) with an ANI value of 82.53%. Finally, genome annotation revealed putative genes encoding pathways for virulence and disease; within this category, we identified homologs of lipoprotein YidQ, the heat shock protein A, and protein YidR and suggest that they play a role in the pathogenicity of strain INIA01.

### Data availability.

The genome sequence is openly available in GenBank of NCBI under the accession number JAPDNC01000000000 (https://www.ncbi.nlm.nih.gov/nuccore/JAPDNC000000000.1/). The associated BioProject, BioSample, and Sequence Read Archive (SRA) numbers are PRJNA893657, SAMN31430933, and SRX18009592, respectively.

## References

[1] Yap MN, Barak JD, Charkowski AO. 2004. Genomic diversity of *Erwinia carotovora* subsp. carotovora and its correlation with virulence. Appl Environ Microbiol 70:3013–3023. doi:10.1128/AEM.70.5.3013-3023.2004. 15128563 PMC404413

[2] Vieira FJ, Nadal-Jimenez P, Teixeira L, Xavier KB. 2020. *Erwinia carotovora* quorum sensing system regulates host-specific virulence factors and development delay in *Drosophila melanogaster*. mBio 11:e01292-20. doi:10.1128/mBio.01292-20. 32576677 PMC7315124

[3] Ares A, Costa J, Joaquim C, Pintado D, Santos D, Messmer MM, Mendes-Moreira PM. 2021. Effect of low-input organic and conventional farming systems on maize rhizosphere in two Portuguese open-pollinated varieties (OPV), “Pigarro” (improved landrace) and “SinPre” (a composite cross population). Front Microbiol 12:636009. doi:10.3389/fmicb.2021.636009. 33717028 PMC7953162

[4] Borruso L, Salomone-Stagni M, Polsinelli I, Schmitt AO, Benini S. 2017. Conservation of Erwinia amylovora pathogenicity-relevant genes among Erwinia genomes. Arch Microbiol 199:1335–1344. doi:10.1007/s00203-017-1409-7. 28695265 PMC5663808

[5] Dickey RS. 1979. Erwinia chrysanthemi: a comparative study of phenotypic properties of strains from several hosts and other Erwinia species. Phytopathology 69:324–329. doi:10.1094/Phyto-69-324.

[6] Schaad NW, Jones JB, Chun W. 2001. Laboratory guide for the identification of plant pathogenic bacteria, 3rd ed. American Phytopathological Society, St. Paul, MN.

[7] Bolger AM, Lohse M, Usadel B. 2014. Trimmomatic: a flexible trimmer for Illumina sequence data. Bioinformatics 30:2114–2120. doi:10.1093/bioinformatics/btu170. 24695404 PMC4103590

[8] Bankevich A, Nurk S, Antipov D, Gurevich AA, Dvorkin M, Kulikov AS, Lesin VM, Nikolenko SI, Pham S, Prjibelski AD, Pyshkin AV, Sirotkin AV, Vyahhi N, Tesler G, Alekseyev MA, Pevzner PA. 2012. SPAdes: a new genome assembly algorithm and its applications to single-cell sequencing. J Comput Biol 19:455–477. doi:10.1089/cmb.2012.0021. 22506599 PMC3342519

[9] Boetzer M, Henkel CV, Jansen HJ, Butler D, Pirovano W. 2011. Scaffolding pre-assembled contigs using SSPACE. Bioinformatics 27:578–579. doi:10.1093/bioinformatics/btq683. 21149342

[10] Boetzer M, Pirovano W. 2012. Toward almost closed genomes with GapFiller. Genome Biol 13:R56. doi:10.1186/gb-2012-13-6-r56. 22731987 PMC3446322

[11] Gurevich A, Saveliev V, Vyahhi N, Tesler G. 2013. QUAST: quality assessment tool for genome assemblies. Bioinformatics 29:1072–1075. doi:10.1093/bioinformatics/btt086. 23422339 PMC3624806

[12] Simao FA, Waterhouse RM, Ioannidis P, Kriventseva EV, Zdobnov EM. 2015. BUSCO online supplementary information: assessing genome assembly and annotation completeness with single-copy orthologs. Bioinformatics 31:3210–3212. doi:10.1093/bioinformatics/btv351. 26059717

[13] Parks DH, Imelfort M, Skennerton CT, Hugenholtz P, Tyson GW. 2015. CheckM: assessing the quality of microbial genomes recovered from isolates, single cells, and metagenomes. Genome Res 25:1043–1055. doi:10.1101/gr.186072.114. 25977477 PMC4484387

[14] Wick RR, Schultz MB, Zobel J, Holt KE. 2015. Bandage: interactive visualization of de novo genome assemblies. Bioinformatics 31:3350–3352. doi:10.1093/bioinformatics/btv383. 26099265 PMC4595904

[15] Aziz RK, Bartels D, Best AA, DeJongh M, Disz T, Edwards RA, Formsma K, Gerdes S, Glass EM, Kubal M, Meyer F, Olsen GJ, Olson R, Osterman AL, Overbeek RA, McNeil LK, Paarmann D, Paczian T, Parrello B, Pusch GD, Reich C, Stevens R, Vassieva O, Vonstein V, Wilke A, Zagnitko O. 2008. The RAST server: Rapid Annotations using Subsystems Technology. BMC Genomics 9:75. doi:10.1186/1471-2164-9-75. 18261238 PMC2265698

[16] Overbeek R, Olson R, Pusch GD, Olsen GJ, Davis JJ, Disz T, Edwards RA, Gerdes S, Parrello B, Shukla M, Vonstein V, Wattam AR, Xia F, Stevens R. 2014. The SEED and the Rapid Annotation of microbial genomes using Subsystems Technology (RAST). Nucleic Acids Res 42:D206–D214. doi:10.1093/nar/gkt1226. 24293654 PMC3965101

[17] Brettin T, Davis JJ, Disz T, Edwards RA, Gerdes S, Olsen GJ, Olson R, Overbeek R, Parrello B, Pusch GD, Shukla M, Thomason JA, III, Stevens R, Vonstein V, Wattam AR, Xia F. 2015. RASTtk: a modular and extensible implementation of the RAST algorithm for building custom annotation pipelines and annotating batches of genomes. Sci Rep 5:8365. doi:10.1038/srep08365. 25666585 PMC4322359

[18] Chaumeil P-A, Mussig AJ, Hugenholtz P, Parks DH. 2019. GTDB-Tk: a toolkit to classify genomes with the Genome Taxonomy Database. Bioinformatics 36:1925–1927. doi:10.1093/bioinformatics/btz848. 31730192 PMC7703759

[19] Jain C, Rodriguez-R LM, Phillippy AM, Konstantinidis KT, Aluru S. 2018. High throughput ANI analysis of 90K prokaryotic genomes reveals clear species boundaries. Nat Commun 9:5114. doi:10.1038/s41467-018-07641-9. 30504855 PMC6269478

[20] Bai Y, Muller DB, Srinivas G, Garrido-Oter R, Potthof E, Rott M, Dombrowski N, Munch PC, Spaepen S, Remus-Emsermann M, Huttel B, McHardy AC, Vorholt JA, Schulze-Lefert P. 2015. Functional overlap of the Arabidopsis leaf and root microbiota. Nature 528:364–369. doi:10.1038/nature16192. 26633631

